# Modelling the risk of food-borne transmission of *Toxocara* spp. to humans

**DOI:** 10.1017/S0950268825000330

**Published:** 2025-06-09

**Authors:** Sara Healy, Eric Morgan, Martha Betson, Joaquin M. Prada

**Affiliations:** 1 School of Veterinary Medicine, University of Surrey, Surrey, UK; 2 Institute for Global Food Security, Queen’s University, Belfast, UK

**Keywords:** food safety, parasite, risk analysis, toxocara, zoonoses

## Abstract

Human toxocariasis is a worldwide parasitic disease caused by zoonotic roundworms of the genus *Toxocara*, which can cause blindness and epilepsy. The aim of this study was to estimate the risk of food-borne transmission of *Toxocara* spp. to humans in the UK by developing mathematical models created in a Bayesian framework. Parameter estimation was based on published experimental studies and field data from southern England, with qPCR Cq values used as a measure of eggs in spinach portions and ELISA optical density data as an indirect measure of larvae in meat portions. The average human risk of *Toxocara* spp. infection, per portion consumed, was estimated as 0.016% (95% CI: 0.000–0.100%) for unwashed leafy vegetables and 0.172% (95% CI: 0.000–0.400%) for undercooked meat. The average proportion of meat portions estimated positive for *Toxocara* spp. larvae was 0.841% (95% CI: 0.300–1.400%), compared to 0.036% (95% CI: 0.000–0.200%) of spinach portions containing larvated *Toxocara* spp. eggs. Overall, the models estimated a low risk of infection with *Toxocara* spp. by consuming these foods. However, given the potentially severe human health consequences of toxocariasis, intervention strategies to reduce environmental contamination with *Toxocara* spp. eggs and correct food preparation are advised.

## Introduction

Human toxocariasis is a worldwide parasitic disease caused by zoonotic roundworms of the genus *Toxocara.* Dogs and cats infected with *Toxocara canis* and *T. cati*, respectively, shed large numbers of parasitic eggs in their faeces [1]. Eggs can then persist for long durations in the environment [2]. If infective eggs are consumed by ‘accidental’ or paratenic host species, including humans, the larvae that hatch out from the ingested eggs subsequently migrate to several different organs in the body, becoming encapsulated in the tissues, and the larvae develop no further [3]. In humans, the migrating larvae and the resulting host response can lead to debilitating disease, including allergic, neurological, and ophthalmic disorders, including vision loss [4]. Globally, 1.4 billion people are estimated to have been exposed to or infected with *Toxocara* spp. [5].

Human infection as a result of inadvertent ingestion of soil contaminated with infective *Toxocara* spp. eggs is widely accepted [6]. In addition, there are a number of published studies which have detected *Toxocara* spp. eggs on unwashed vegetable crops in Iran, Libya, Turkey, and the UK [7–12], and these could pose a public health risk when eaten. Grazing animals can also become infected by consuming infective *Toxocara* spp. eggs from the pasture, and larvae were detected in their tissues in a handful of studies in Norway, Japan, Italy, and Iran [10, 13–16]. This represents an infection risk to humans if meat is consumed undercooked (i.e. cooked for less than 2 minutes at 70°C as recommended by the UK Food Standards Agency (FSA)) [17] or raw [18]. Despite the potentially severe clinical manifestations of human toxocariasis and the confirmed presence of *Toxocara* spp. on vegetable produce and within meat tissues, the actual risk of transmission of this parasite to humans via the food chain remains unknown.

The aim of this study was to estimate the risk of food-borne transmission of *Toxocara* spp. to humans in the UK by developing mathematical models created in a Bayesian framework.

## Methods

**Figure 1.**
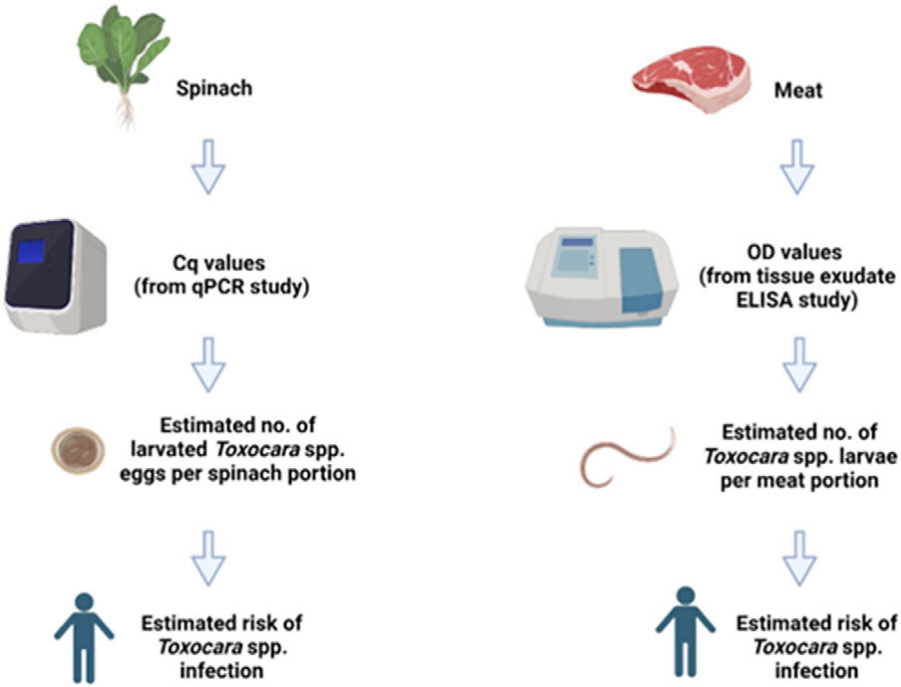
The conceptual models used in the study. Cq: Quantification Cycle (obtained from qPCR data), OD = Optical Density (obtained from spectrophotometer data), No. = Number. Image created using Biorender.com.

Two models were developed, one for spinach consumption and one for meat consumption. Spinach was selected to represent a leafy variety of vegetable, which can be more susceptible to contamination with soil containing parasite eggs due to their uneven surface and broad leaves, which tend to trap more soil during cultivation [19], and it is often consumed without prior cooking, thus posing a potentially greater risk to consumers. A diagram outlining the conceptual models is shown in Figure 1. Briefly, using previously published field data on vegetable contamination with *Toxocara* spp. eggs and anti-*Toxocara* spp. antibody prevalence in meat juice [20, 21], we used qPCR data to estimate the likely number of larvated eggs (for spinach) and ELISA data to estimate the likely number of larvae in tissue (for meat). The models estimated the proportion (%) of portions positive for *Toxocara* spp. larvated eggs/larvae, the number of eggs/larvae per portion, and the human risk (%) of *Toxocara* spp. infection per portion. For the purpose of the models, one portion of spinach was 300g, which was based on the original sample size of the field spinach collected in a previous study by Healy et al. [20]. The meat portion weights (i.e. the average reported weight of meat consumed per portion [22]) varied depending on the species (beef, lamb, or pork), as outlined in Table 1.

**Table 1.** Model data used for the weight of each portion of meat and the number of edible portions of each tissue type available for each species. The typical portion weights consumed for each meat type and animal species shown in the table were obtained from Patel and Mills [22]

Species	Tissue type	Portion weight (g)	Total tissue weight (g) per animal	Total portions per animal
Sheep	Muscle	90	20000	222
Sheep	Liver	40	520	13
Pig	Muscle	120	90000	750
Pig	Liver	50	1710	34
Cow	Muscle	140	120000	857
Cow	Liver	40	3100	77.5

### Exposure from leafy vegetables

The number of infective eggs (each containing an infective larva) expected to be found in unwashed leafy vegetables needed to be estimated; however, there are limited data available. We first used data from a study linking known quantities of non-larvated *T. canis* eggs to qPCR-generated quantification cycle (Cq) values [7] to calibrate a logistic function that captures the expected Cq value from a given number of non-embryonated eggs (Equation 1). We assume that the Cq value measured is related to the number of eggs (neggs) in the sample through Equation 1, with some Gaussian (normally distributed) noise, Ƭ.(1)

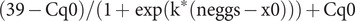

Cq values were capped at 39.0 to minimize the risk of false-positive results, as this is a late point in the plateau stage of the amplification cycle, above which it is assumed that any fluorescence detected is generated by nonspecific amplification of background nucleic acids. Thus, values over 39.0 were classed as negative samples, and this value sets the upper limit for equation (1). Cq0 is the lowest value of the function and represents the cycles expected when the number of eggs is high (tending to infinity). k captures the steepness of the curve and x0 the midpoint (inflection point) of the function in terms of the number of eggs.

**Figure 2.**
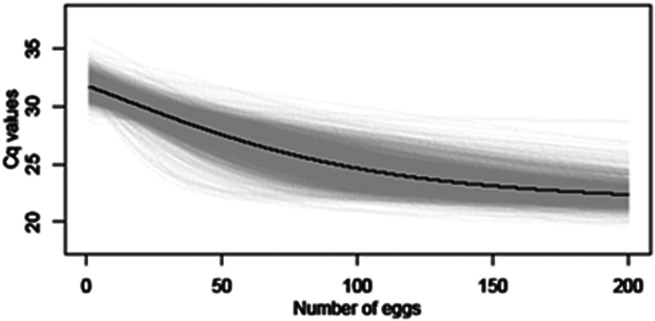
Average logistic curve (black line) and individual trajectory outputs (grey lines) estimated linking number of non-larvated eggs with Cq values using qPCR.

The model was fitted in the Bayesian framework using JAGS (Just Another Gibbs Sampler) in R with the ‘runjags’ package [23]. Uninformative priors were used for Ƭ and k; for x0 and Cq0, uniform distributions ranging from 0–200 and 18–22.26 were used, respectively. A range up to 200 eggs was chosen for x0, as this was the point at which the Cq values appeared to plateau in preliminary model runs, with no substantial change in Cq value with increased egg numbers. A range of 18–22.26 was selected for Cq0, as 18 was the lowest Cq value observed in preliminary model runs, and 22.26 was the Cq value observed experimentally for large numbers (500) of unembryonated *T. canis* eggs. Two chains of 10000 samples were drawn after 5000 iterations of burn-in with a thinning of 10. The fitted logistic curve is shown in Figure 2.

In the paper by Jarosz et al. [24], the mean and standard deviation in the Cq value were reported for two known quantities of larvated eggs. Given that the data were too limited to calibrate a model, we assumed a logistic function with the same shape as the one fitted above to the non-larvated eggs but ‘shifted down’ (i.e. a known quantity of larvated eggs will have a lower Cq value than the same quantity of non-larvated eggs). The quantity by which it was ‘shifted’ was calculated in each model run, taking into account the uncertainty in the logistic function fitted above and the variation in the Cq values from the paper [24].

To estimate the number of infective (larvated) eggs in commonly accessible leafy vegetables, we used the distribution of Cq values reported by Healy et al. from the South of England as a representative sample [20]. Then, the following steps were carried out in each model run repeat, following a similar approach to the model fitting presented in previously published modelling studies [25, 26].

1. Values were drawn from a uniform distribution for the logistic function and shift, as well as field Cq values drawn from those reported by Healy et al. [20].
2. The empirical standard deviation for the expected Cq values given different numbers of larvated and non-larvated eggs (from 0 to 200) was calculated.
3. The distance (error) between the field Cq value and each of the expected Cq values was calculated as the square-root of the differences squared.
4. 25 out of the 400 (6.25%) possible options with the lowest error were selected (the value of 25 was selected as an arbitrary cut-off number). Here, each option is a specific number of eggs (larvated or non-larvated).
5. Each option was weighted using the Epanechnikov kernel with a normalizing constant equal to 1 [27]
6. The number of larvated eggs for that field sample with a specific Cq value was a single draw from the 25 options selected taking into account their weight.

Each model simulation selected 1000 samples and estimated the proportion of those that have larvated eggs and the total number of larvated eggs across all samples. This was repeated 2000 times to generate the 95% credible intervals.

### Exposure from meat products

For the meat model, the estimated number of infective larvae within meat tissues was again evaluated using an exponential model as outlined above, with some modifications outlined below:

1. Optical density (OD) readings were obtained following ELISA analysis of tissue exudate samples [21]. Using this data in conjunction with published data from rodent studies of tissue larval burdens with their associated serum ELISA OD readings [28, 29] and individual rodent tissue larval count data with the corresponding serum ELISA OD readings for each animal (obtained from Dr. Lescano, personal communication), the number of larvae present in meat tissues based on tissue exudate OD readings was estimated. An assumption of the model is that serum ELISA OD values are equivalent to tissue exudate ELISA OD values.
2. The data were used to estimate the probability of a meat type being consumed using the volumes of beef, lamb, and pork purchased in the UK per year are outlined in Table 2. Data for the annual volume of different meat types consumed in the UK [30], and the average meat portion sizes consumed [22] were used in the model to estimate the level of exposure to tissue larvae per individual consuming one portion of meat per day.

As before, each model simulation selected 1000 samples but, in this case, it estimated the proportion of samples that have larvae and the total number of larvae across all samples. This was repeated 2000 times to generate the 95% credible intervals. The outputs of both the spinach and meat models were used to develop the next stage of the model: estimating the risk of human infection.

**Table 2.** Data used in the model to provide an estimate of public consumption of each meat type in the UK per year and the probability of the meat type being consumed (probability = volume for species/total volume). Data shown for volumes of meat purchased were obtained from the Agriculture and Horticulture Development Board [30]

Meat type	Volume (kg) purchased for consumption in the UK per annum (million)	Probability of meat type being consumed (%)
Beef	122	35.5
Lamb	15	4.4
Pork	207	60.2
Total	344	

### Risk estimation

To estimate the risk to humans consuming spinach containing infective *Toxocara* spp. eggs or meat tissues containing infective *Toxocara* spp. larvae, an exponential dose–response model was calibrated using the optim function in R. The data were taken from a *Toxocara* spp. rodent dose–response study undertaken in 1995 by Havasiová-Reiterová et al. [31] that reports the dosage of *Toxocara* eggs, the number of animals consequently infected with *Toxocara* larvae in their tissues and the number of larvae found within each tissue type.

A summary of the data used for both models and the sources of data are outlined in Table 3. For each model, the exposure data obtained in Section titles “Exposure from leafy vegetables” and “Exposure from meat products” (i.e. the average proportion (%) of portions positive for *Toxocara* spp. larvated eggs/larvae and the average number of eggs/larvae per portion) in conjunction with the dose–response model developed in Section title “Risk estimation” was used to estimate the average risk per individual consuming a portion of unwashed spinach or undercooked meat. All model code is available at: https://github.com/Sara-Vet/Food-Risk-Assessment-Toxocara.git

**Table 3.** A summary of the data extracted from each external source used for the models developed in the current study, listed in order of appearance

Reference name and citation	Data extracted from source	How data was used
Jarosz et al. [24]	Mean Cq^a^ values obtained by qPCR for embryonated *Toxocara canis* eggs (two batches; *n* = 7 and *n* = 12 per group).	Data used for spinach model to weight Cq values into two groups; infective and non-infective eggs. Only infective (larvated) eggs pose a risk to human health.
Moura et al. [28]	Mean numbers of larvae within the livers of mice (*n* = 20 per group) following oral inoculation with 50, 100, 250, 500, or 1000 *T. canis* eggs, 45 days post-infection, and serum ELISA optical density (OD) readings for different tissue larval burdens	Data used for the meat model to estimate the number of larvae in the tissues for a given infective dose of *Toxocara* eggs. The serum ELISA OD values were used to estimate the number of tissue larvae for different tissue exudate ELISA OD values
Lescano et al. [29]	Mean numbers of larvae within the liver, lungs, kidneys, brain, eyes and carcass of mice (*n* = 10 per group) following oral inoculation with 300 embryonated *T. canis* eggs, sampled 120 days post-infection. Individual mouse data for tissue larval burden and corresponding serum ELISA OD was obtained by personal communication with the author	Data used in conjunction with the Moura et al. (2020) data outlined above to estimate the number of tissue larvae for a given infective dose of *Toxocara* eggs. The serum ELISA OD values were used in conjunction with the Moura et al. (2020) data above, to estimate the number of tissue larvae for different tissue exudate ELISA OD values
**Agriculture and Horticulture Development Board** (Markets and Prices) [30]	The volume (kg) of each meat type purchased for consumption in the UK per annum (million).	Data used in the model to provide an estimate of public consumption of each meat type in the UK, per year. The probability of a particular meat type being consumed was calculated and used to estimate the exposure of humans to larvae in meat.
Patel and Mills [22]	The average portion weights (g) consumed for different types of meat.	Data used for the meat model to estimate the amount of tissue ingested in a portion, and hence the exposure of larvae to humans.
Havasiová-Reiterová et al. [31]	The mean numbers of larvae recovered from the liver and muscles of mice (*n* = 22 per group) and the mean larval recovery rate from the tissues, 70 days after oral inoculation with either 5 or 7 infective *T. canis* or *T. cati* eggs.	Data for infective egg doses and corresponding larval recovery rates from rodent tissues used to develop a dose-response curve for the spinach model.

a Cq = Quantification cycle (qPCR readings)

## Results

The estimated risk of an individual becoming infected with *Toxocara* spp. from consuming a portion of undercooked meat was 0.172% per portion and 0.016% per portion of spinach; this represents a 10-fold higher estimated risk for meat consumption compared to the consumption of unwashed spinach. The average proportion of meat portions that were positive for *Toxocara* larvae was estimated at 0.841%, compared to spinach, in which 0.036% of portions contained infective eggs. This represents a 23-fold higher probability of finding *Toxocara* spp. larvae in undercooked meat tissues compared to eggs on spinach crops. For meat tissues, the estimated number of larvae per portion was 0.859, compared to 0.097 larvated eggs per portion of spinach; this equates to an 8-fold difference between these food types.

**Figure 3.**
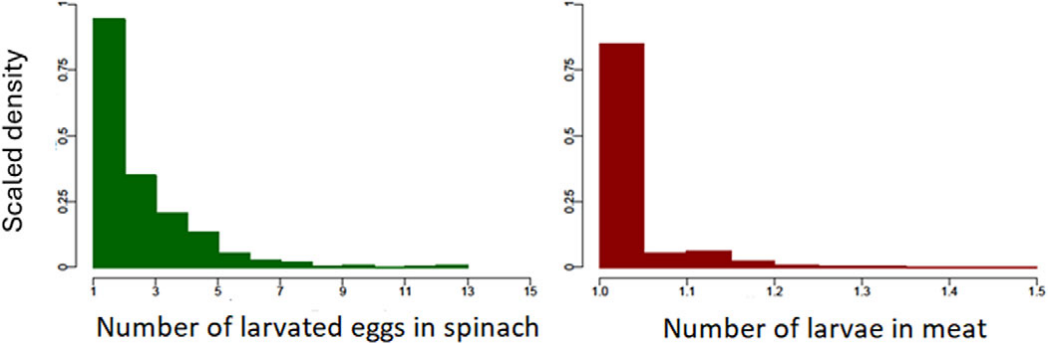
Scaled density histograms showing the estimated number of larvated *Toxocara* spp. eggs and *Toxocara* spp. larvae in each positive portion of spinach and meat respectively, based on 2000 model iterations. Each bar of the histogram represents the relative probability of finding a data point for a given number of eggs or larvae.

The outputs of both the spinach and meat models are summarized in Table 4. For the samples estimated by the model to be positive for eggs or larvae, the estimated average number of larvated *Toxocara* spp. eggs per positive portion of spinach ranges from 1 to 13, while *Toxocara* spp. larvae in positive meat portions are, on average, low and close to one, as shown in Figure 3.

**Table 4.** Summary of the outputs of the spinach and meat models. (a) The average proportion (%) of portions of unwashed spinach/undercooked meat positive for *Toxocara* spp. eggs/larvae (95% CI) (b) The average number of *Toxocara* spp. eggs or larvae per portion of unwashed spinach/undercooked meat respectively. (c) The estimated risk (%) of human *Toxocara* spp. infections resulting from the ingestion of unwashed spinach and undercooked meat tissues, per portion consumed

Food type	(A) Average proportion (%) of portions positive for *Toxocara* spp. eggs/larvae (95% CI)	(B) Average number of eggs/larvae per portion (95% CI)	(C) Average human risk (%) of *Toxocara* spp. infection, per portion consumed (95% CI)
Spinach	0.036 (0.000–0.200)	0.097 (0.000–0.600)	0.016 (0.000–0.100)
Meat	0.841 (0.300–1.400)	0.859 (0.300–1.500)	0.172 (0.000–0.400)

## Discussion

This study aimed to estimate the probability of *Toxocara* spp. larvated eggs or larvae being present within spinach (representing leafy vegetables) and meat and the risk of human *Toxocara* spp. infection from consuming these food products using a mathematical modelling approach. Overall, the model outputs estimated the proportion of unwashed leafy vegetables or undercooked meat tissues positive for larvated *Toxocara* spp. eggs or larvae as being low, and with a low average number of larvated eggs/larvae per positive portion. A very small number of spinach portions were estimated to have higher numbers of larvated eggs: as many as 13, compared to a maximum of 1.5 larvae within a single meat portion. Given that the response to a single *Toxocara* spp. larvae can lead to pathology in humans [32], in the small number of cases where multiple larvated eggs are present in a serving of spinach, consuming these portions could pose a greater risk of harm to humans if not washed before ingestion. However, given the small proportion of samples positive for *Toxocara* spp. overall, the models estimated a low average risk of human infection from consuming these food products.

As this is the first study to investigate the risk of food-borne *Toxocara* spp. transmission, and only limited food prevalence studies exist in the literature for this parasite, it is difficult to evaluate how well the findings of this study represent food-borne *Toxocara* spp. risk in general. In the case of *Trichinella*, another food-borne parasite most commonly associated with pork products, risk assessment modelling undertaken by Franssen et al. in 2018 reported an estimated average number of human trichinosis cases as < 0.002 (range 0.000–0.007), per year, for the whole of the European Union (EU). This figure was based on *Trichinella* testing at meat inspection being undertaken and pigs originating from farms with high levels of biosecurity. Without meat inspection, this figure rose slightly to <0.010 (range 0.001–0.023) per year, and if pigs were reared on farms without biosecurity controls and no meat inspection was undertaken, the estimated number of trichinosis infections in the EU was 59,443 (95% CI, 52-837–66, 360) per year [33]. These findings highlight the importance of farm-level biosecurity controls and meat inspection in reducing the risk of food-borne parasites, something which is not currently undertaken in the case of *Toxocara* spp.

Whilst the presence of helminth eggs on vegetable produce have been reported quite widely in the literature [34], studies to quantitively evaluate the risk of transmission of helminths to humans via the consumption of vegetables are lacking. Leafy vegetables, in particular, appear to be susceptible to contamination with parasitic eggs and could pose a higher risk of parasitic infection if consumed without prior washing; risk evaluation for food types such as these would especially be of benefit to inform food safety practices [12, 35]. There are more risk analysis studies available in the literature for protist organisms in food products. For example, a recent Brazilian study reported an estimated mean incidence of *Toxoplasma gondii* of up to 9.68 infections per 1000 people, per day, for leafy vegetables, 6.32 for beef and 2.03 for pork [36], although the authors recognize that assumptions made with respect to oocyst and bradyzoite viability may have overestimated the values they obtained. In a similar study assessing the risk of *T. gondii* in meat to consumers in China, for the centre of the country, an estimated mean incidence of 13 infections per 1000 people, per day, was reported for pork meat, 3.9 infections for beef, and 7 infections for small ruminant meat [37]. However, as before, the authors do recognize that the modelled outputs are likely to be an overestimate of the true risk to public health. As for the current study, a lack of available data and the use of rodent study data for dose–response modelling is a limitation. Further research building upon the findings of the current study to assess the risk of food-borne helminths to consumers, including measures of the impact of crop cultivation and food hygiene intervention measures on the risk of transmission, is urgently needed.

In this study, the data selected as prevalence inputs for the spinach model were obtained from a field study of *Toxocara* spp. DNA prevalence from field-grown spinach crops [20]. An assumption for the spinach model was that the DNA present on the spinach samples represented viable eggs (larvated or not). However, it is possible that DNA from damaged, non-viable eggs was detected by the RT-PCR assay, which could have resulted in over estimation of the exposure and risk outputs of the model. In addition, for a given Cq value, it is not possible to know for sure whether the DNA present originated from a large number of non-larvated eggs, a smaller number of larvated eggs (each containing a greater concentration of DNA due to larval development) or indeed a mixture of both larvated and non-larvated eggs. As only larvated eggs pose a risk of human infection, to try and manage this limitation, previously published Ct values for known quantities of larvated eggs were incorporated into the model [24] to differentiate the outputs into infective vs. non-infective eggs. Furthermore, the size of the spinach portions used to develop the model in this study were 300 g each. This portion weight was based on the size of the field samples obtained to obtain the Cq values in a previous study [20] but, in reality, represents quite a large volume compared to the typical portion weight for this vegetable of 80 g [38]. The large portion weight used for spinach in this study may have influenced estimations of the number of eggs a consumer would be exposed to daily. For meat, consumption estimates were based on UK averages (Table 2), but in reality, consumer preferences for certain meat types and portion sizes would result in a greater heterogeneity of risk. In addition, as a dose–response model was used to estimate risk in this study, its non-linear nature would mean any change in portion size would have a non-linear effect on risk (e.g. a reduction of the portion size by half would not equate to half the risk).

The data selected as prevalence inputs for the meat model were obtained from an ELISA study of anti-*Toxocara* antibodies within meat tissue exudates, with 27.7% of samples in the study testing positive [21]. Whilst no larvae were detected within the meat samples analysed in that study, the presence of antibodies in quite a high number of samples demonstrates that exposure of meat-producing animals to *Toxocara* spp. from the environment is common. An important assumption for this study was that an animal with anti-*Toxocara* spp. antibodies within its tissue exudates will have viable larvae within its tissues and thus pose some risk of transmission to humans via consumption of its meat. There is no certainty around this, however, as an animal may have cleared the infection with residual antibodies being detected despite no viable larvae within its tissues, and thus the number of meat portions with viable larvae may be lower than predicted. In addition, the OD values in the model were scaled down to 2.0 to align with the rodent data used, but the data obtained for OD readings for meat tissue exudates extended to an upper limit of 4.0 [21]. Due to a lack of available data on tissue larval counts and corresponding tissue exudate OD values in food animal species, a linear scaling approach was taken, and it also had to be assumed that there was an equal distribution of larvae within liver and muscle tissues with no difference in larval tissue burdens between different food animal species. In addition, the dose–response data used for both models were obtained from experimental rodent *Toxocara* spp. studies. The possibility of variations in the dose–response of *Toxocara* spp. in food animal species and humans compared to rodents cannot be ruled out. For example, a recent study by Poulsen et al. suggested that a porcine model of *Toxocara* infection could be superior to rodent models due to closer similarities between pigs and humans in their size, body weight, immune response, liver physiology, and metabolic function [39]. Future research to address these limitations, incorporating more data for livestock species as it becomes available, is required to increase the reliability of estimates for *Toxocara* spp. transmission to humans via the food chain.

The models developed in this study assessed the probability of exposure to *Toxocara* spp. in undercooked meat and unwashed spinach and the associated risk of human infection in the south of England. However, the impact of intervention measures to reduce the risk of developing toxocariasis, such as washing spinach and freezing or thorough cooking of meat tissues (i.e. cooking for 2 minutes at 70^o^C as recommended by the FSA [17]) was not incorporated into either model due to a lack of availability of quantitative data. An Iranian study undertaken in 2020 showed that washing vegetables in 200 ppm calcium hypochlorite solution was the most effective approach to remove zoonotic parasites, with less than 1% of vegetables found to be contaminated post-treatment [35]. However, the approaches outlined in this study are not commonly implemented in the UK, with potable water alone most frequently used for vegetable washing. Incorporating data for calcium hypochlorite treatment of vegetables would have a significant impact on the estimated risk of *Toxocara* infection from leafy vegetables to a negligible level, but the model outputs would be unlikely to reflect the estimated risk to UK consumers. While there are no available data on the impact of cooking temperature or duration on the viability of larvae in typical meat cuts, a limited number of published studies have shown that chilling can reduce the infectivity of *Toxocara* spp. larvae in meat [40] and delay embryonation of eggs [41], with freezing to −20°C found to lead to death of *Toxocara* spp. larvae [42] and reduced egg viability [43]. Future research to assess the public health risks associated with *Toxocara* spp. in food products and the impact of intervention measures, such as cooking, on the risk of transmission is warranted.

The models in this study only evaluated *Toxocara* spp. in spinach and meat tissues from pigs, sheep, and cattle. However, these are not the only food types which can transmit this parasite, and other types of fruits, vegetables, and meat types (such as poultry and game) may be implicated [10]. In addition, the data used for the models were from spinach and meat sampled from the south of England, and different prevalence levels may be appropriate in other regions of the UK and other countries. The models also did not incorporate the risk of acquiring *Toxocara* spp. directly from the environment, which is a widely accepted transmission route to humans [6], and therefore do not compare risk from food with these other routes.

Both *Toxocara canis* and *T. cati* are known to be zoonotic to humans, but these species could not be differentiated in this study due to a lack of available data. For example, the tissue exudate study data used for the meat model used an ELISA-based assay which is not able to differentiate between *T. canis* and *T. cati* due to the homology between the excretory-secretory antigens of these species [44]. Our knowledge regarding the differences in human disease pathogenesis between these two species is currently lacking [45], but improved diagnostic techniques and increased data availability regarding the risks implicated for each species will strengthen the reliability of future risk assessment models.

In conclusion, this study produced quantified estimates of ingesting infective *Toxocara* stages in animal- and plant-derived foods and provides a useful framework, which could be utilized for future risk-assessment studies to evaluate different systems, contexts, and potential interventions to uphold food safety and public health. Results indicate a lower risk of being exposed to larvated *Toxocara* spp. eggs within unwashed spinach compared to larvae within undercooked meat products, and the risk of acquiring a *Toxocara* spp. infection from the consumption of these food products was predicted to be small, particularly for spinach. Given the potentially severe clinical consequences of *Toxocara* spp. infection on human health, however, mitigation strategies to reduce environmental contamination with *Toxocara* spp. eggs, including on farmland used both to grow food and keep grazing animals, and consumer intervention around correct preparation and cooking of food are prudent to protect public health. Further research to increase understanding of food-borne transmission of *Toxocara* spp. is required to inform public health policy and protect consumers.

## Data Availability

The data that support the findings of this study and the model code used are openly available at: https://github.com/Sara-Vet/Food-Risk-Assessment-Toxocara.git
